# EMERGENCY DEPARTMENT USE AND HEALTH CARE EXPENDITURES AMONG OLDER ADULTS AT END-OF-LIFE

**DOI:** 10.1093/geroni/igae098.0130

**Published:** 2024-12-31

**Authors:** Cameron Gettel, Courtney Kitchen, Craig Rothenberg, Yuxiao Song, Arjun Venkatesh, Susan Hastings

**Affiliations:** Yale University School of Medicine, New Haven, Connecticut, United States; Yale University School of Medicine, New Haven, Connecticut, United States; Yale University School of Medicine, New Haven, Connecticut, United States; Yale University School of Medicine, New Haven, Connecticut, United States; Yale University School of Medicine, New Haven, Connecticut, United States; Durham VA, Durham, North Carolina, United States

## Abstract

Emergency department (ED) visits at end-of-life (EOL) may cause financial strain to older adults and serve as a marker of inadequate access to community services and health care. We sought to examine EOL ED use, total health care spending, and out-of-pocket (OOP) spending among Medicare beneficiaries. Using data from the Medicare Current Beneficiary Survey, we conducted a pooled cross-sectional analysis including Medicare beneficiaries aged 65 years and older with a date of death between July 1, 2015 and December 31, 2021. Our primary outcomes were ED visits, total healthcare spending, and OOP healthcare spending in the 7, 30, 90, and 180 days preceding death. We compared total and OOP healthcare costs for those with at least 1 ED visit prior to death vs. without any ED visits within each timeframe and estimated a series of zero-inflated negative binomial regression models to identify associations between key beneficiary characteristics and the primary outcomes. Among 3,812 older adult decedents, 610 (16%), 1,207 (31.7%), 1,582 (41.5%), and 1,787 (46.9%) Medicare beneficiaries, respectively, had ED visits in the final 7, 30, 90, and 180 days of life. For Medicare beneficiaries with at least one ED visit in the final 30 days of life, the median total and OOP costs were respectively $12,366 and $160, compared respectively to $160 and $0 for those without any ED visits (P <0.001 for both comparisons). These estimates of ED use and costs during the terminal phase of life highlight the importance of appropriate and timely EOL care.

